# α-Lipoic Acid Reduces Hypertension and Increases Baroreflex Sensitivity in Renovascular Hypertensive Rats

**DOI:** 10.3390/molecules171113357

**Published:** 2012-11-09

**Authors:** Thyago M. Queiroz, Drielle D. Guimarães, Leônidas G. Mendes-Junior, Valdir A. Braga

**Affiliations:** Biotechnology Center, Federal University of Paraiba, João Pessoa, PB 58.051-900, Brazil; Email: thyagoqueiroz@gmail.com (T.M.Q.); drielle_dantas@hotmail.com (D.D.G.); lmjunior05@hotmail.com (L.G.M.-J.)

**Keywords:** 2K1C, blood pressure, reactive oxygen species, lipoic acid, parasympathetic, sympathetic

## Abstract

Renovascular hypertension has robust effects on control of blood pressure, including an impairment in baroreflex mechanisms, which involves oxidative stress. Although α-lipoic acid (LA) has been described as a potent antioxidant, its effect on renovascular hypertension and baroreflex sensitivity (BRS) has not been investigated. In the present study we analyzed the effects caused by chronic treatment with LA on blood pressure, heart rate and baroreflex sensitivity (sympathetic and parasympathetic components) in renovascular hypertensive rats. Male Wistar rats underwent 2-Kidney-1-Clip (2K1C) or sham surgery and were maintained untouched for four weeks to develop hypertension. Four weeks post-surgery, rats were treated with LA (60 mg/kg) or saline for 14 days orally. On the 15th day mean arterial pressure (MAP) and heart rate (HR) were recorded. In addition, baroreflex sensitivity test using phenylephrine (8 µg/kg, i.v.) and sodium nitroprusside (25 µg/kg, i.v.) was performed. Chronic treatment with LA decreased blood pressure in hypertensive animals; however, no significant changes in baseline HR were observed. Regarding baroreflex, LA treatment increased the sensitivity of both the sympathetic and parasympathetic components. All parameters studied were not affected by treatment with LA in normotensive animals. Our data suggest that chronic treatment with LA promotes antihypertensive effect and improves baroreflex sensitivity in rats with renovascular hypertension.

## 1. Introduction

**Figure 1:**
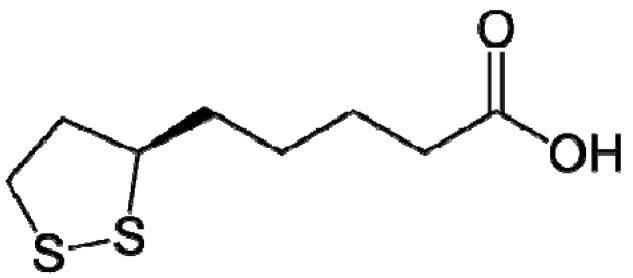
Chemical structure of (*R*)-α-lipoic acid.

α-Lipoic acid (LA), also known as 1,2-dithiolane-3-pentanoic acid or thioctic acid, is a disulfide antioxidant with one chiral center that exists in both *R*- and *S*-enantiomeric forms. However, only (*R)*-LA is conjugated to conserved lysine residues in an amide linkage, thus making this isoform essential as a cofactor in biological systems (Figure 1) [1,2]. Among its properties, α-lipoic acid functions as a cofactor in the multienzyme complexes that catalyze the oxidative decarboxylation of α-ketoglutarate and pyruvate dehydrogenase, critical enzymes in ATP production [1,3]. LA is also absorbed intact from dietary sources, and it can be accumulated in tissues. There is evidence that orally supplied LA might not be used as a metabolic cofactor but instead, elicits important activities with potential therapeutic value [2].

LA and its reduced form, dihydrolipoic acid, reduce oxidative stress by scavenging a number of free radicals in both membrane and aqueous domains [4]. In addition, this thiol antioxidant, which holds a metal chelating capability, is able to regenerate endogenous antioxidants such as vitamins C and E and also increases nitric oxide (NO) activity [5,6,7].

Increased oxidative stress and associated oxidative damage are considered mediators of vascular injury in cardiovascular pathologies, including hypertension and atherosclerosis [8,9]. The relationship between oxidative stress and hypertension has been demonstrated in several models of experimental hypertension. For example, increased reactive oxygen species (ROS), including superoxide (O_2_^•−^), hydroxyl (OH^•^) and peroxyl radicals (ROO^•^) or hydrogen peroxide (H_2_O_2_), formation precedes the development of hypertension in spontaneously hypertensive rats (SHR) and in angiotensin II (Ang II)-infused mice [10,11,12,13]. In this latter model, the ROS production is dependent on NADH/NADPH, a plasma membrane-bound protein that, when activated by binding of Ang II in AT_1_ receptors, produces ROS [14,15].

Hypertension and cardiovascular diseases are the leading cause of death in developed and developing countries [16], causing a great impact on global human health. In an attempt to reduce this impact, in recent decades several research groups have worked extensively to improve the treatment of cardiovascular diseases including the discovery of new therapy strategies and drugs [17,18]. Among these, we can highlight the use of an antioxidant therapy such as ROS scavengers and vitamins, superoxide dismutase (SOD) mimetics or NADPH oxidase inhibitors such as apocynin, to attenuate or prevents the development of hypertension [19,20,21,22].

It is well established that alterations in blood pressure is direct associated with changes in the baroreceptor reflex function. Baroreceptors situated in the aortic arch and carotid sinuses sense variations in blood pressure and trigger reflex autonomic adjustments that buffer alterations in blood pressure. In pathological conditions such as hypertension, there is impairment in the autonomic regulation of blood pressure resulting in damage in baroreflex sensitivity [23,24].

In view of the fact that reactive oxygen species seems to play an important role in autonomic control during health and disease and that effects of antioxidant therapy involved in baroreflex control are not completely understood, in the present study we tested the hypothesis that chronic administration of α-lipoic acid, a key antioxidant, would promote an antihypertensive effect and improve baroreflex sensitivity in renovascular hypertensive rats.

## 2. Results and Discussion

### 2.1. Renal Artery Clipping Induces Hypertension without Affecting Heart Rate

**Figure 2:**
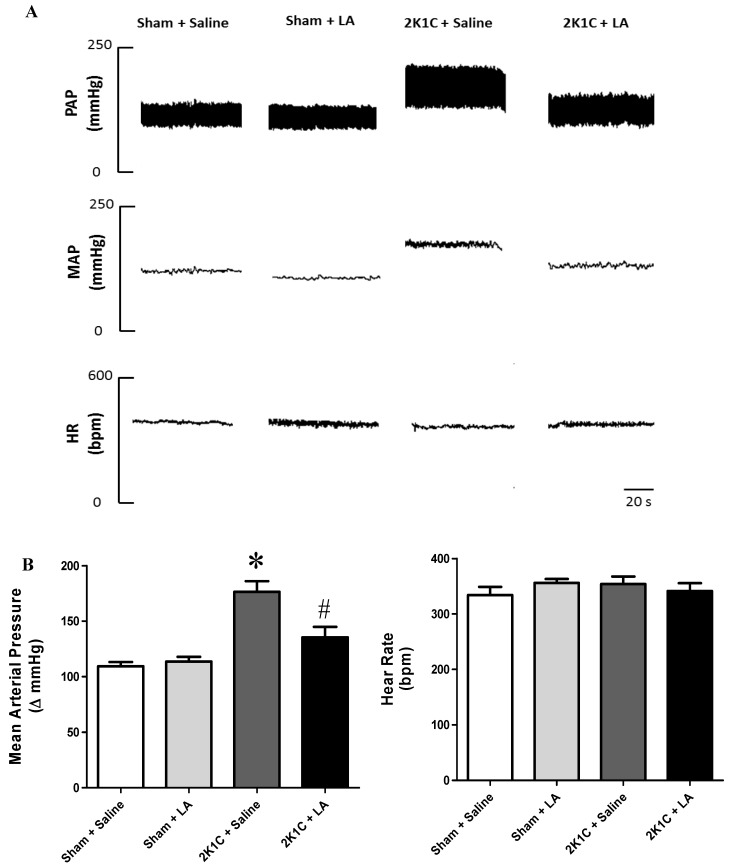
(**A**) Representative tracings showing the changes in pulse arterial pressure (PAP, mmHg), mean arterial pressure (MAP, mmHg), and heart rate (HR, bpm) of a rat from Sham + Saline; Sham + LA; 2K1C + Saline and 2K1C + LA groups four weeks after clipping the right renal artery. (**B**) MAP and HR values of Sham + Saline (n = 6, open bar); Sham + LA (n = 6, light grey bar); 2K1C + Saline (n = 6, dark grey bar) and 2K1C + LA (n = 6, black bar) groups. * *p* < 0.05, when compared to Sham + Saline group. # *p* < 0.05, when compared to 2K1C + Saline group.

Four weeks after clipping of the kidney, rats from the 2K1C + Saline group (n = 8) were hypertensive when compared to the sham-operated group (n = 8), (176.5 ± 9.6 *vs*. 109.3 ± 3.8 mmHg, respectively, *p* < 0.05). However in the 2K1C + LA group (n = 7) we did not observe differences in relation to the Sham + LA group (n = 6), (133.5 ± 9.3 *vs*. 113.6 ± 4.2 mmHg, respectively). In addition rats from the 2K1C + LA group presented a reduction in blood pressure when compared to 2K1C + saline group (133.5 ± 9.3 *vs*. 176.5 ± 9.6 mmHg, respectively), as shown in the representative tracings and in the group data in Figure 2A,B.

These results corroborate other studies which have been reported that common antioxidants for clinical use and experimental trials such as ascorbic acid (AAC) and *N*-acetylcysteine (NAC) were able to delay or prevent the development of different types of hypertension. When compared the intensity of the reduction in blood pressure, we observed that the hypotensive effect promoted by LA was similar to values found from the previous studies [13,25]. Indeed, known to scavenge hydroxyl radicals, singlet oxygen, hydrogen peroxide, hypochlorous acid, peroxynitrite and nitric oxide. The present investigation is the first description of antihypertensive effect of LA in the renovascular hypertension model. This finding has important clinical significance because the Goldblatt 2K1C experimental model of renovascular hypertension is characterized by excess of the activity of the rennin-angiotensin-aldosterone system (RAAS) and increased circulating levels of angiotensin-II, which is identified as the main mediator of the mechanisms involved in the essential hypertension [26]. These include vasoconstriction, induction of vascular smooth muscle cell growth [27,28], stimulation of proto-oncogene expression [29,30,31], modulation of myocardial hypertrophy and fibrosis [32,33,34,35] and modulation of ventricular remodeling after myocardial infarction [15]. Several evidences suggest that a key mechanism by which angiotensin-II influences blood pressure is via its ability to stimulate the production of reactive oxygen species [12,14], mainly superoxide anion [36,37], via activation of NADPH oxidase.

NADPH oxidase is an enzyme composed of two membrane-bound subunits (gp91phox and p22phox), cytoplasmic subunits (p40phox, p47phox, and p67phox), and the small G protein Rac1a [38]. After activation of AT-1 receptors by angiotensin-II, the cytoplasmic subunits bind to the membrane subunits and activate the enzyme, resulting in the intracellular production of superoxide. The increase of superoxide leads to changes in ion channels, particularly calcium and potassium channels, altering neuronal firing properties in areas of the brain such as the rostral ventrolateral medulla (RVLM) resulting in increase in sympathetic nerve activity and increase in blood pressure [39]. Besides of blood pressure measurement, heart rate was also evaluated in this study. Interestingly, chronic administration of α-lipoic acid did alter baseline heart rate in normotensive or hypertensive rats as illustrated in Figure 2.

### 2.2. Chronic Administration of LA Restores Baroreflex Sensitivity in Renovascular Hypertension

In order to investigate the baroreflex sensitivity, after baseline MAP and HR recordings, animals received intravenous injections of phenylephrine (Phe, 8 µg/kg) and sodium nitroprusside (SNP, 25 µg/kg) to determine the baroreceptor reflex responses. Acute administration of Phe was used to evaluate the parasympathetic component and SNP to evaluate the sympathetic autonomic component of the baroreflex.

**Figure 3:**
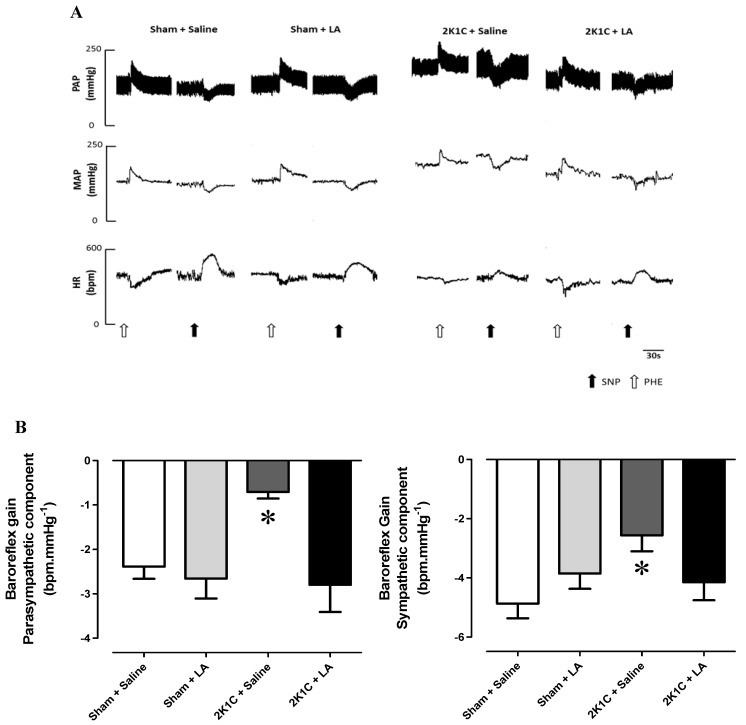
Baroreflex sensitivity test. (**A**) Representative tracings from each of the four groups (Sham + Saline; Sham + LA; 2K1C + Saline and 2K1C + LA) showing the changes in pulse arterial pressure (PAP, mmHg), mean arterial pressure (MAP, mmHg), and heart rate (HR, bpm) in response to phenylephrine (black arrows) and sodium nitroprusside (open arrows). (**B**) Values for baroreflex sensitivity (bpm.mmHg^−1^) determined by the modified Oxford method using intravenous injection of NPS and Phe in Sham + Saline (n = 6, open bar); Sham + LA (n = 6, light grey bar); 2K1C + Saline (n = 6, dark grey bar) and 2K1C + LA (n = 6, black bar) groups. * *p* < 0.05, when compared to Sham + Saline group.

Representative traces showing the changes in heart rate in response to baroreflex activation are illustrated in Figure 3A. Rats from the 2K1C + Saline group presented a reduction in baroreflex sensitivity in both parasympathetic and sympathetic components when compared to the Sham + Saline group (−0.70 ± 0.1 *vs.* −2.38 ± 0.2 and −2.56 ± 0.5 *vs*. −4.86 ± 0.5 bpm.mmHg^−1^, respectively, *p* < 0.05, n = 8) as shown in Figure 3B. In 2K1C rats, chronic treatment of LA (60 mg/Kg, v.o.), restored the depressed baroreflex sensitivity to values found by sham-operated rats treated with LA in both parasympathetic and sympathetic component of baroreflex system (−2.80 ± 0.6 *vs*. −2.61 ± 0.4 and −4.14 ± 0.6 *vs*. −3.85 ± 0.5 bpm.mm Hg^−1^, *p* < 0.05, n = 8).

There is convincing experimental evidence that oxidative stress modulates baroreflex function in health and disease [40,41]. For example, administration of exogenous free radical scavengers, such as superoxide dismutase and catalase, to the carotid sinus in rabbits with experimentally induced atherosclerosis improves the depressed baroreflex function [42]. These results suggest an inhibitory role of reactive oxygen species on carotid baroreceptors. In addition, acute intravenous infusion of ascorbic acid, a well-known antioxidant, increases baroreflex sensitivity in patients with cardiac dysfunction [43,44].

According to these related studies, as well as in the present investigation, administration of antioxidants had no effect on baroreflex function in normotensive animals [42,43], suggesting that anti-oxidant therapy in the absence of oxidative stress has no influence on baroreflex sensitivity. Mutually, the results from the present study and those from previous findings support the insight that oxidative stress reduces baroreflex sensitivity and that anti-oxidant therapy, such as α-lipoic acid, could restore it.

Although our data suggest that the antioxidant-mediated improvement in baroreflex sensitivity observed in 2K1C rats is probably due to increase in autonomic function, it is not possible to determine the exact mechanism at which LA exerts its positive influence on baroreflex function in renovascular hypertensive rats. Evidence from other experimental studies has suggested that diminished baroreflex sensitivity is caused by endothelial dysfunction (ED) [44]. Because decreasing the synthesis and release of NO is the main factor to promote ED, studies has been shown that LA improves endothelial NO synthesis and thus improving endothelial function [45]. In particular, in experimentally induced endothelial dysfunction, the decreased prostacyclin and increased thromboxane concentrations are associated with reduced baroreflex impulses from the carotid artery [44]. Moreover, experimental evidence strongly suggests a direct suppressive influence of ROS on baroreceptors (*i.e.*, a peripheral site of action) [42]. The enzyme NADPH oxidase has been suggested as a possible target to increase ROS generation. A number of drugs used for the treatment of hypertension, hypercholesterolaemia and coronary artery disease such as the statins, AT1 (angiotensin II receptor type 1) antagonists and ACE inhibitors have been shown to decrease NADPH oxidase-derived superoxide and ROS production [46,47,48]. These therapies could repair the pro-oxidants levels and reestablish the regular conditions of the baroreflex sensitivity as well as cardiovascular parameters in the renovascular hypertension.

## 3. Experimental

### 3.1. Animals

Twenty-seven adult male Wistar rats (280–330 g) were housed in conditions of controlled temperature (21 ± 1 °C) and exposed to a 12 h light-dark cycle with free access to food (Labina^®^, Purina, São Paulo, Brazil) and tap water. All procedures described in the present study are in agreement with Institutional Animal Care and Use Committee of the Federal University of Paraiba (CEPA/LTF protocol nº 0305/09).

### 3.2. Surgical Procedures

Rats underwent surgical procedures in order to develop renovascular hypertension (2K1C model) as described by Botelho-Ono *et al.* [13]. Briefly, under combined ketamine and xylazine anesthesia (75 and 10 mg/kg, i.p., respectively), a midline abdominal incision was made. The right renal artery was exposed and isolated over a short segment by blunt dissection. A U-shaped silver clip (0.2 mm internal diameter) was placed over the vessel at a site proximal to the abdominal aorta and the wound closed and sutured. A sham procedure, which entailed the entire surgery with the exception of renal artery clipping, served as control. After the procedures, rats returned to their home cages and remained untouched for six weeks in order to develop hypertension.

### 3.3. Blood Pressure and Heart Rate Recordings

After six weeks, rats were anesthetized with ketamine and xylazine (75 and 10 mg/kg, i.p., respectively), polyethylene catheters inserted into the lower abdominal aorta and inferior vena cava through left femoral artery and vein, respectively. Both catheters were filled with heparinized saline, tunneled subcutaneously, exteriorized and sutured at the dorsal surface of the neck. Twenty-four hours after surgical procedures, experiments were performed in conscious rats as previously described [49]. Blood pressure and heart rate were recorded using a pressure transducer (MLT0380/D, ADInstruments, Sydney, Australia) and connected to a computer (Mikro-tip blood pressure system, ADInstruments) running the LabChart software (ADInstruments).

### 3.4. Baroreflex Sensitivity Test

For baroreflex sensitivity studies, animals were divided in four different groups: Sham + Saline and Sham + LA, 2K1C + Saline, 2K1C + LA. Four weeks after the clip implantation, animals were treated with a daily dose of Lipoic Acid (60 mg/kg, v.o.) or Saline for fourteen days. At the day of the experiment, following baseline blood pressure and heart rate recordings, baroreflex was activated using classical vasoactive drugs (modified Oxford Method, Braga *et al.*) [50] before and after bolus acute administration of vasoactive drugs: Phenylephrine (8 µg/kg) and sodium nitroprusside (25 µg/kg). A 15-minute interval as allowed between phenylephrine and sodium nitroprusside injections. Reflex changes in heart rate produced by vasoactive drugs administration were quantified and plotted as changes in heart rate over changes in mean arterial pressure (ΔHR/ΔMAP) as described by Braga *et al.* [50] All data from each group were analyzed by linear regression using the GraphPad Prism software and the slope of the linear regression yield baroreflex gain for each group.

### 3.5. Statistical Analyses

Results are expressed as mean ± SEM. Data were analyzed by Student’s t test or two-way repeated measures analysis of variance (ANOVA) followed by a Dunnett post-test for multiple comparisons whenever appropriate. All statistical analyses were performed using GraphPad Prism (v. 5.0, GraphPad Software, Inc.). Statistical significance was defined as *p* < 0.05.

## 4. Conclusions

In summary, our results suggest that antioxidant therapy by chronic treatment with α-lipoic acid reduces hypertension and improves baroreflex sensitivity in rats with renovascular hypertension. Despite the precise site of action where the chronic anti-oxidant treatment produces its beneficial effects remains unknown, it seems to involve both peripheral and central mechanisms, since LA has an amphipathic structure and thus can cross the blood-brain barrier.
